# As Clear as Mud? Determining the Diversity and Prevalence of Prophages in the Draft Genomes of Estuarine Isolates of *Clostridium difficile*

**DOI:** 10.1093/gbe/evv094

**Published:** 2015-05-27

**Authors:** Katherine R. Hargreaves, James R. Otieno, Anisha Thanki, Matthew J. Blades, Andrew D. Millard, Hilary P. Browne, Trevor D. Lawley, Martha R.J. Clokie

**Affiliations:** ^1^Department of Infection, Immunity and Inflammation, University of Leicester, United Kingdom; ^2^Department of Ecology and Evolutionary Biology, University of Arizona; ^3^KEMRI-Wellcome Trust Research Programme, Kilifi, Kenya; ^4^Bioinformatics and Biostatistics Analysis Support Hub (BBASH), Core Biotechnology Services, University of Leicester, United Kingdom; ^5^Microbiology & Infection, Warwick Medical School, University of Warwick, Coventry, United Kingdom; ^6^Microbial Pathogenesis Laboratory, Wellcome Trust Sanger Institute, Hinxton, United Kingdom

**Keywords:** pathogen genome evolution, prophage, mobilome, *Clostridium difficile*, bacteriophage, virulence

## Abstract

The bacterium *Clostridium difficile* is a significant cause of nosocomial infections worldwide. The pathogenic success of this organism can be attributed to its flexible genome which is characterized by the exchange of mobile genetic elements, and by ongoing genome evolution. Despite its pathogenic status, *C. difficile* can also be carried asymptomatically, and has been isolated from natural environments such as water and sediments where multiple strain types (ribotypes) are found in close proximity. These include ribotypes which are associated with disease, as well as those that are less commonly isolated from patients. Little is known about the genomic content of strains in such reservoirs in the natural environment. In this study, draft genomes have been generated for 13 *C. difficile* isolates from estuarine sediments including clinically relevant and environmental associated types. To identify the genetic diversity within this strain collection, whole-genome comparisons were performed using the assemblies. The strains are highly genetically diverse with regards to the *C. difficile* “mobilome,” which includes transposons and prophage elements. We identified a novel transposon-like element in two R078 isolates. Multiple, related and unrelated, prophages were detected in isolates across ribotype groups, including two novel prophage elements and those related to the transducing phage φC2. The susceptibility of these isolates to lytic phage infection was tested using a panel of characterized phages found from the same locality. In conclusion, estuarine sediments are a source of genetically diverse *C. difficile* strains with a complex network of prophages, which could contribute to the emergence of new strains in clinics.

## Introduction

The pathogenic bacterium *Clostridium difficile* causes nosocomial infections in humans, but can also be carried asymptomatically, which has implications for its disease transmission (Eyre et al. 2013). The bacterium may move through the food chain, as it has reservoirs in livestock and has been isolated from contaminated food stuffs (see review [Gould and Limbago 2010]). *Clostridium difficile* is also present in natural environments such as river water (Zidaric et al. 2010), soil (Al Saif and Brazier 1996; Higazi et al. 2011), seawater (Al Saif and Brazier 1996; Pasquale et al. 2011), and estuarine sediments (Hargreaves et al. 2013). Studies suggest that the presence of *C. difficile* in the natural environment is transient with recovery of the strains variable between sites and time, but sustained with the species present at sites across a location (Zidaric et al. 2010; Higazi et al. 2011; Hargreaves et al. 2013). Although transmission between livestock and human strains has been observed (He et al. 2013), it is unclear whether natural environments represent an epidemiological “dead end” or could represent an additional source of circulating *C. difficile* strains.

The *C. difficile* genome has been described as “highly plastic” (Sebaihia et al. 2006) and contains multiple mobile genetic elements (MGEs) including several transposons and prophages, and the presence or absence of which occur between different strains (Stabler et al. 2006, 2009; He et al. 2010; Monot et al. 2011; Cairns et al. 2012; Corver et al. 2012; Mullany et al. 2015). Several transposons have been characterized; including and Tn*916*, which confer tetracycline resistance; Tn*4452*a and Tn*4453*b, which confer chloramphenicol resistance and Tn*5398* which carries the antibiotic resistance gene *ermB*. These examples of *C. difficile* transposons demonstrate how carriage of MGEs can promote bacterial fitness in a clinical setting (reviewed in Mullany et al. 2015). Their contribution to the pathogenicity of strains is less established, but strains lacking the *skin*^Cd^ element have reduced levels of sporulation (Haraldsen and Sonenshein 2003). The *C. difficile skin*^Cd^ element (sigKintervening element) has evolved from a decayed prophage element which could influence sporulation, but lost the ability to excise (Mullany et al. 2015). Studies using *C. difficile* molecular genetics have revealed the function of several key genes linked to its pathogenicity and ability to cause disease. Strains can produce up to at least three toxins; TcdA and TcdB which are encoded by genes in the Pathogenicity Locus, and the binary toxin (CDT), encoded by *cdtA* and *cdtB*, which are regulated by *cdtR* (Rupnik et al. 2009). Additional virulence genes relate to colonization and survival both within host, and when outside the gut, such as those involved in flagella biosynthesis, sporulation, and quorum sensing (Tasteyre et al. 2001; Dingle et al. 2011; Deakin et al. 2012; Martin et al. 2013; Barketi-Klai et al. 2014).

We have previously described the distribution and biology of *C. difficile* strains isolated from sites located throughout an estuarine system, in Hampshire, United Kingdom (Hargreaves et al. 2013). The established method used to type *C. difficile* is based on the polymerase chain reaction (PCR) amplification of the variable intergenic region between the 16S rRNA and 23S rRNA genes to generate ribotype profiles (O’Neill et al. 1996). The estuarine isolates include ribotypes which are prevalent within UK clinical samples, such as R027 and R078, as well as ribotype groups that are less frequently reported from patient samples, such as R010 (Wilcox et al. 2012). We used transmission electron microscopy (TEM) to show that a high proportion of the isolates contain viable prophages and PCR targeting-specific myovirus genes, which suggested that there is a significant prophage diversity within these strains (Hargreaves et al. 2013).

*Clostridium difficile* phages have been described which have unusual characteristics which could affect their bacterial host phenotype. These include altering toxin transcription and production during infection with the myoviruses φC2 and ΦCD119, and the siphovirus φCD38-2 (Goh, Chang, et al. 2005; Govind et al. 2009; Sekulovic et al. 2011), and the presence of gene homologs to the accessory gene regulator (*agr*) quorum sensing system in the myovirus phiCDHM1 (Hargreaves, Kropinski, et al. 2014). To date, all sequenced *C. difficile* phages are temperate (Govind et al. 2006; Goh et al. 2007; Mayer et al. 2008; Horgan et al. 2010; Sekulovic et al. 2011; Meessen-Pinard et al. 2012; Hargreaves, Kropinski, et al. 2014) and can therefore introduce new genes into susceptible *C. difficile* strains as prophages. Phage can perform generalized transduction during infection by erroneously packaging host bacterial chromosome in place of the phage genome. In this species, the phage φC2 was shown to transfer antibiotic resistance between strains in this manner (Goh et al. 2013).

The capacity for strains to acquire novel genetic material by phage infection may also be influenced by the carriage of related prophages already within the bacterial chromosome, through immunity by superinfection, and as is the case for *C. difficile*, the CRISPR/Cas (Clusters of Regularly Interspaced Palindromic Repeats) system which confers immunity against invading phages, dependent on complimentary spacer sequences present in arrays to that of the foreign DNA (Barrangou et al. 2007). *Clostridium difficile* prophages can contain CRISPR arrays that are expressed and processed (Sebaihia et al. 2006; Soutourina et al. 2013), and spacers match to other phage genome sequences, which could in theory limit genetic exchange (Hargreaves, Flores, et al. 2014).

From the previously isolated estuarine strains, we selected 13 isolates that represent the ribotype diversity from this source. We performed whole-genome sequencing to determine the genetic diversity present in these strains and to establish the potential of the mobilome to impact upon strain evolution. Draft genome assembly, annotation, and comparative analysis has identified variable content between strains with regards to the virulence factors, including toxin gene carriage, the *agr* loci, and the regions encoding genes involved in flagella biosynthesis and S-layer production. The comparative genomic analysis demonstrated clear differences in the carriage of MGEs, including conjugative and mobilizable transposons. We also identified a novel transposon-like element in two R078 isolates. Our data confirm the high carriage of prophage sequences which are related to known *C. difficile* phages, and we have identified two new prophage types that appear to be related to siphoviruses.

## Materials and Methods

### Genomic DNA Extraction and Sequencing

Genomic DNA was lysed from *C. difficile* isolates grown overnight by means of lysozyme, proteinase K and RNase A, and was purified using standard phenol–chloroform methods. Whole-genome sequencing was performed using the Solexa Illumina HiSeq2000 platform from multiplexed paired-end libraries (100 bp read length, 150–250 bp insert size) generating an average of 2.1 million reads per sample.

### Draft Genome Assembly and Annotation

Raw read data preprocessing included, removal of adapter sequences using Cutadapt (Martin 2011), 3′ read trimming and filtering of low quality reads by FASTX-Toolkit[74][74][74][76][76][76][75] (accessed at http://hannonlab.cshl.edu/fastx_toolkit/ ) and read quality assessment using FastQC (accessed at http://www.bioinformatics.babraham.ac.uk/projects/fastqc/)[75][75][75][77][77][77][76].

Reads were assembled using Velvet (Zerbino and Birney 2008), parameters were optimized using the Velvet-optimizer script [64]. Contig alignments were performed with the draft genome assemblies in MAUVE (Darling et al. 2004) using the progressiveMAUVE algorithm (Darling et al. 2010). The contigs for each assembly were then reordered in reference to the genome sequence of *C. difficile* strain CD630 (accession NC_009089) and realigned. To improve contig reordering, the draft genome of isolate CD105HS8 (R027) was then reordered to CD196 (NC_013315), and those of the two isolates belonging to R078; CD105HS27 and CD105HS26, were each reordered in reference to M120 (FN665653). Realignment of the genome assemblies was then performed to visualize the optimization and final architecture of the draft genomes.

For strain accession numbers, see table 1*.* Unaligned contigs are retained and positioned at the end of the alignments. The draft genomes were then annotated with Prokka v1.7 (Seamann 2014) using a custom database containing CD630 CDSs and visualized using Artemis v15.0.0 (Rutherford et al. 2000). Further sequence analysis was performed using BLASTn and BLASTp against the nonredundant nucleotide and whole-genome shotgun databases at the NCBI and aa sequence searches against the Pfam database, both accessed online. Sequence alignments were performed using Clustal Omega (Sievers et al. 2011) accessed online.

**Table 1:** Draft Genome Information

Isolate	Ribotype	Number of Contigs	N50 Contig Length	Total Sequence Length	%GC	Average Coverage	%mapped Reads	Assembly Accession	Raw File Accession
CD105HS9	R010	156	155,576	4,150,738	29.59	105.58	87.20	ERS515332	ERS688197
CD105HS16	R010	173	138,599	4,153,415	29.65	141.56	87.62	ERS515337	ERS688200
CD105HS22	R220	130	162,540	4,105,138	29.13	104.71	93.75	ERS515341	ERS688200
CD105HS6	R220	251	55,953	4,078,848	29.83	106.89	94.11	ERS515331	ERS688195
CD105HS27	R078	515	19,847	3,985,572	31.41	89.74	89.95	ERS515340	ERS688198
CD105HS26	R078	614	14,579	3,959,653	31.4	108.74	91.00	ERS515334	ERS688194
CD105HS8	R027	94	113,960	4,073,110	29.32	98.16	93.67	ERS515333	ERS688192
CD105HS1	R012	254	65,061	4,153,492	31.16	112.2	89.83	ERS515329	ERS688189
CD105HS19	R031	193	123,830	3,995,146	30.03	127	96.81	ERS515336	ERS688193
CD105HS4	R014	185	86,936	4,252,466	28.83	71.26	85.14	ERS515330	ERS688190
CD105HS7	R002	412	29,552	4,189,668	30	73.14	81.57	ERS515335	ERS688199
CD105HS10	R005	230	81,567	4,254,080	29.57	59.65	87.99	ERS515339	ERS688191
CD105HS12	R001	159	96,828	4,051,221	29.44	89.55	94.80	ERS515338	ERS688196

### Comparative Genomic Analyses

The draft genomes were compared against CD630 using Blast Ring Image Generator (BRIG) v0.95 based on BLASTn (Alikhan et al. 2011). Multi-FASTA files were used a references to assess the variation at virulence-linked loci, including the PaLoc; each individual PaLoc genes *tcdABCER* from CD630, as well as *cdtR* from CD630 and *cdtAB* from CD196 in BRIG, the flagella biosynthesis regions F1, F2 and F3 from CD630, and the S-layer locus from CD630. Genome sequence comparison was also performed using ACT v13.0.0 (Carver et al. 2005). Phylogenetic analysis was performed on the *agrB* genes from the draft genomes in MEGAv6 (Tamura et al. 2013), which were first aligned using MUSCLE, analyzed using the inbuilt model test, and ML analysis performed using the Jones–Taylor–Thornton (JTT) model with uniform rates, using all sites and Nearest-Neighbor-Interchange. Antibiotic resistance profiles were generated using the Genome Annotation tool at the ARDB (Liu and Pop 2009). Two-way ANI was calculated from draft genomes and reference genomes using the ANI calculator (available at http://enve-omics.ce.gatech.edu/ani/index) using ANI options of minimum length 700 bp, minimum identity 70%, minimum alignments 50, window size 1,000 bp, and step size 200 bp.

### Prophage Prediction and Analysis

A multi-FASTA file containing the publically available *C. difficile* phage genomes of φC2 (NC_009231), ΦCD119 (NC_007917), phiCDHM1 (HG531805), ϕCD27 (NC_011398), ΦMMP02 (NC_019421), ΦCD6356 (NC_015262), φCD38-2 (NC_015568), and ΦMMP04 (NC_019422) was used as a reference to blast against the draft genomes in BRIG. Prophage regions were identified in the draft genomes using PHAST (Zhou et al. 2011). Following manual checking and examination, novel prophage regions were reannotated using FGENES (Softberry Inc., USA). Annotation of CDSs was based on results of aa sequence searches against the Pfam database (accessed at http://pfam.xfam.org/) and using BLASTp against the NCBI nr protein database. Prophage genomes were visualized using EasyFig v2.1 (Sullivan et al. 2011). Phylogenetic analysis of the phage endolysin genes of the novel prophage elements, the published phage genomes previously mentioned, and *Clostridium* phages phiCDHM11 (HG798901), phiCDHM13 (HG796225), phiCDHM14 (LK985321), phiCDHM19 (LK985322) were included, as well as the *Clostridium perfringens* phage phiSM101 (CP000315) endolysin gene as an outgroup. This analysis was performed using MEGAv6 following alignment with the inbuilt MUSCLE and model test, and ML analysis was performed using the JTT model with bootstrap test of 500 reiterations.

### Bacterial Culture and Antibiotic Resistance Testing

Bacterial isolates were routinely grown on 1% bacterial agar Brain Heart Infusion (BHI, Oxoid, UK) plates under anaerobic conditions in a Don Whitely Scientific Chamber at 37 °C. Colonies were grown overnight from stocks kept at −80 °C in 80% glycerol. Strains were streaked onto 1% agar Luria Broth plates and with 150 µl of single antibiotic stocks added to the surface of the plate under sterile conditions to produce tetracycline 10 and 30 µg ml^−^^1^, and erythromycin at 10 and 20 µg ml^−^^1^ plates. Plates were examined for growth following overnight incubation.

### Phage Isolation, Characterization, and Bacterial Susceptibility

Phages were isolated from enriched cultures from estuarine sediment samples following spot assays on to indicator hosts CD105HE1, CD105HS1, and CD105LC1 from our strain collection and strain ZZV09-1622 kindly provided by Maja Rupnik, University of Maribor, Slovenia. Phages were also induced from environmental isolates and tested for lytic infection on these strains. Phages were purified following at least three rounds of replication from single plaques, their particle morphology assessed by TEM, with samples stained with 1% uranyl acetate, and phage genome sizes determined by pulsed field gel electrophoresis (PFGE). PFGE was performed as described (Nale et al. 2012). Isolates were screened for their susceptibility to a morphologically diverse panel of phages; these included medium-sized myoviruses phiCDHM1 (previously described; Hargreaves, Kropinski, et al. 2014), phiCDHM3 (this study), phiCDHM19 (Hargreaves, Flores, et al. 2014), and phiCDHM23 (this study); long-tailed myoviruses phiCDHM2, phiCDHM4, phiCDHM5, and phiCDHM6 (this study); small myoviruses phiCDHM9 (this study), phiCDHM11, phiCDHM13, phiCDHM14 (Hargreaves, Flores, et al. 2014), and a siphoviruses phiCDHS1 (this study). Susceptibility was performed in triplicate using double agar overlay method to produce a bacterial lawn for each isolate using 250 µl overnight culture in fastidious anaerobic broth (Bioconnections, UK) added to 2.5 ml 0.4% agar BHI and 10 µl of phage stock spotted on once set. Plates were incubated under anaerobic conditions overnight and checked for lytic clearing or turbid clearing.

## Results

### Whole-Genome Sequencing, Assembly, and Comparison

The 13 isolates were sequenced and the draft genome assemblies range in total lengths from 3,959,653 to 4,254,080 bp and had a GC% content range of 28.83–31.41. The average coverage was at a minimum of 59.65× and a maximum of 141.56× per genome. Assembly statistics are provided alongside strain accession and ribotype information in table 1.

The isolates represent ten ribotypes; R001, R002, R005, R010, R014, R012, R220, R027, R031, and R078, as determined previously (Hargreaves et al. 2013). The overall genome similarity was assessed by their average nucleotide identity (ANI) as a measure of relatedness (Goris et al. 2007). The isolates ranged in identity to one another from 96.36% to 100% (supplementary table S1, Supplementary Material online). Within the ribotype groups, the isolates shared greater than 99% identity, with the R220 isolates at 99.91%, R010 at 99.9%, with the environmental R078 environmental isolates 99.34%. The R078 isolates shared an ANI value to the reference M120 of 99.6% and 99.5%, respectively, for CD105HS26 and CD105HS27. The environmental R027 shared 99.05% identity to the clinical CD196 strain and CD105HS1 to CD630 shared 99.78%. The lowest ANI scores were generally between R078 isolates to any other ribotype or isolate, ranging from 96.36 to 96.77%. The lowest identity shared between the remainder of the isolates, regardless of ribotype was 98.95% between CD105HS1 and CD105HS8. One-way ANOVA (analysis of variance) found that ANI identity variance differed significantly between ribotypes and within ribotypes (*P* = 0.025).

### Genome Sequence BLASTn Comparisons Highlight Divergent Regions between Isolates

**Fig. 1.—:**
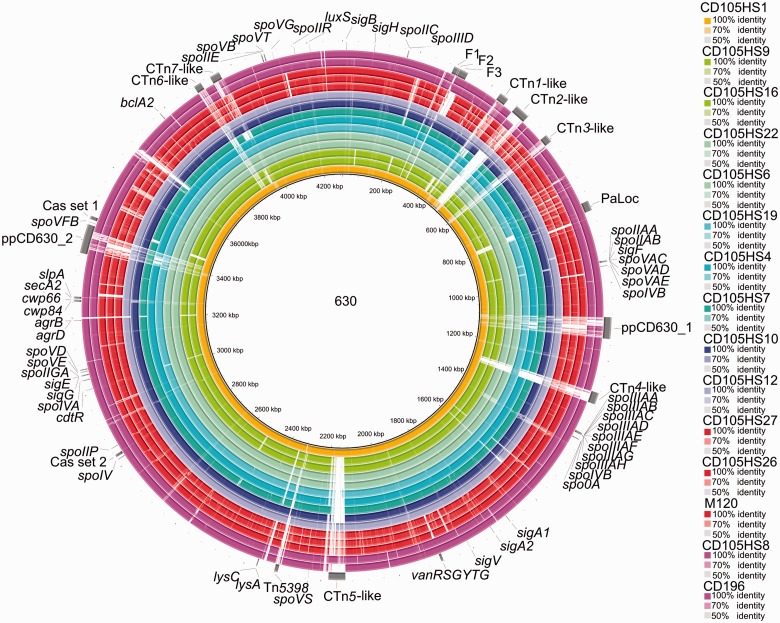
Whole-genome comparisons between environmental *C. difficile* and reference strain CD630. Composite genome comparison figure generated using BRIG from performing a BLASTn analysis for each of the 13 isolates, strains CD196 and M120 to reference genome CD630. On rings from the inside to the outside tracks are: CD105HS1 (R012), CD105HS9 (R010), CD105HS16 (R010), CD105HS22 (R220), CD105HS6 (R220), CD105HS19 (R031), CD105HS4 (R014), CD105HS7 (R002), CD105HS10 (R005), CD105HS12 (R001), CD105HS27 (R078), CD105HS26 (R078), M120 (R078), CD105HS8 (R027), CD196 (R027) and gene features of interest from CD630 annotation which are labeled. The color correlates to ribotype group, for example, R078 are red and R027 are magenta and intensity to sequence similarity %. Gapped regions indicate the absence or low similarity between the genomes. Several regions of divergence are evident. Some of these are conserved between several isolates in the data set and others are specific to one isolate or a ribotype. Seven conjugative transposons are marked from the CD630 annotation and the two prophages, which are highly divergence across the analysis.

To assess regions of genetic similarity between the draft genomes, sequence comparisons were performed with reference to the type strain CD630 and visualized in BRIGv0.95 using BLASTn (Alikhan et al. 2011). The draft genomes are arranged in concentric rings and sequence similarity is shown by intensity of color shading. Included in the analysis are the reference genome sequences of CD196 (R027) and M120 (R078) (fig. 1). The genes that have been annotated on figure 1 include those encoding components of the flagella biosynthesis, sporulation, and quorum sensing pathways. Gaps in the shading indicate absence of homologous sequences. Several of these gapped regions occur in all isolates and are located where transposons and prophages are in CD630.

Comparisons of the environmental R078 isolates to M120 were performed in BRIG, using the genome sequences of M120, CD105HS27, and CD105HS26 each as references (supplementary fig. S1, Supplementary Material online). Further comparison in ACT revealed the presence of a novel transposon-like element containing several predicted transposases, transposon proteins and recombinases, in CD105HS27 and CD105HS26, which is not present in M120 (supplementary fig. S2, Supplementary Material online). This element is 104,221 nt in length on node 1523 in the draft genome of CD105HS27. It is shared with CD106HS26, but is fragmented, with 21,383 nt length in a corresponding location with additional related sequence in unordered contigs at the end of the assembly. The element contains coding sequences (CDSs) encoding putative sporulation factors, cell surface proteins, and a LexA repressor, in addition to DNA replication and regulatory proteins (see supplementary table S2, Supplementary Material online, for list of predicted gene functions). Searches using BLASTn against the NCBI (National Centre for Biotechnology Information) nt/nr and WGS databases revealed it to also be present in a human isolate of *C. difficile* E1 (R126) from Austria (Kurka et al. 2014) with query coverage of 95% and identity of 99%, and next best BLASTn hit to an Italian human strain, T5 (R126), (Kurka et al. 2014) with 43% coverage and 99% identity. In addition to being found in *C. difficile,* there were regions of sequence similarity to other *Clostridiales,* with the best BLAST hit to *Clostridium saccharolyticum* with a total coverage of 31% and identity of 99%*.*

### Toxin and Virulence Associated Genes Carriage

*Clostridium difficile* strains belong to different toxin types according to their toxin gene carriage (Rupnik 2010). Previously, the toxin gene carriage was determined for these isolates based on PCR amplification of conserved regions of the *tcdB*, *tcdA*,** and *cdtAB* genes (Hargreaves et al. 2013). In order to gain a comprehensive view of the toxin gene diversity within these strains, we examined their presence and diversity from the draft genome data.

Each genome assembly was queried using BLASTn against a multi-FASTA file containing the sequences of *tcdABERC* and *cdtR* from CD630, and *cdtAB* from CD196 in BRIG (supplementary fig. S3, Supplementary Material online). We found that toxin gene carriage typically correlates to results of the PCR assay where known (Hargreaves et al. 2013). However, in the cases of the R078 isolates, some of these genes appear highly fragmented, truncated or missing due to contig breaks in the assembly. This was manually inspected using MAUVE and Artemis (data not shown). The PaLoc is known to have undergone multiple gain, exchange, and loss events throughout the species (Dingle et al. 2014). Due to contig assembly gaps conclusions about its evolution cannot be made from our data, but the transposon Tn*6218*, which can interrupt the PaLoc (Dingle et al. 2014), was not identified.

Although both CD105HS9 and CD105HS16 are atoxigenic, as confirmed by PCR and the BRIG analysis, the BLASTn analysis identified sequences in both isolates that are similar to *tcdE.* These sequences are likely to be derived from phage-encoded holins based on the analysis of adjacent genes*.* Although in this case the BLASTn approach produced a “false positive,” in contrast, it resulted in visualizing the fragmented toxin gene content of the R078 isolates against the reference genes which were missed in the genome annotation, highlighting the use of this approach.

To assess other key genetic determinants of phenotypic relevance in the draft genomes, the F1-3 flagella regions and the S-layer locus which encodes SlpA were also examined. Of the flagella genes, the most variance was observed in the F2 region, but these regions were also interrupted in several draft genomes by contig gaps (supplementary fig. S4, Supplementary Material online). In the SlpA locus, the *slpA* gene displayed the most divergence between isolates (supplementary fig. S5, Supplementary Material online).

*Clostridium difficile* strains have also been found to contain multiple *agr* loci (Sebaihia et al. 2006; Stabler et al. 2009; Hargreaves, Kropinski, et al. 2014). The loci present in the environmental isolates were investigated using maximum-likelihood (ML) phylogenetic analysis of the *agrB* at the aa level with genes from CD630, M120, and two from CD196; CD196_3141 and CD196_2593 (supplementary fig. S6, Supplementary Material online). The sequences cluster into three groups, each supported by a bootstrap value of greater than 85. For nine of the isolates, *agrB* genes were identified which fall into clusters for the *agr1* and *agr2* loci, with four isolates that have genes in the *agr1* locus only. Finally, four isolates have *agrB* sequences that cluster with the *agr3* locus including three R078 isolates.

Antibiotic resistance gene profiles were generated from each draft assembly, using ARDB—Antibiotic Resistance Genes Database. Predicted bacitracin resistance was identified for all isolates, aminoglycoside resistance for five isolates, erythromycin resistance for three isolates, and tetracycline resistance in two isolates above the cut off threshold of *E* value 0.05 and %identity less than 80.

Although *C. difficile* is resistant to several antibiotics [43], it has been shown that carriage of resistance genes does not necessarily confer resistance, as has been previously shown with respect to the case of the vancomycin cassette, *vanGCd* [44]. The antibiotic MICs of these isolates were previously determined for vancomycin, metronidazole, clindamycin, and ciprofloxacin (Hargreaves et al. 2013). In this study, we assessed antibiotic resistance to erythromycin and tetracycline, following growth on agar plates. Six isolates grew on 20 µg ml^−^^1^ erythromycin, which included all three isolates with genes predicted for erythromycin resistance (supplementary table S3, Supplementary Material online). Both R078 isolates were found to be resistant to tetracycline at 10 µg ml^−^^1^. One of these isolates was also erythromycin resistant, and carried the predicted genes, whereas the other isolate does not and showed no resistance.

### Shared and Novel Prophage Regions within the Environmental Isolates

The species is known to be highly lysogenic and prophage carriage was examined within these draft genomes using known *C. difficile* phage sequences as a multireference input in BRIG and the prophage prediction software PHAST to determine novel types.

**Fig. 2.—:**
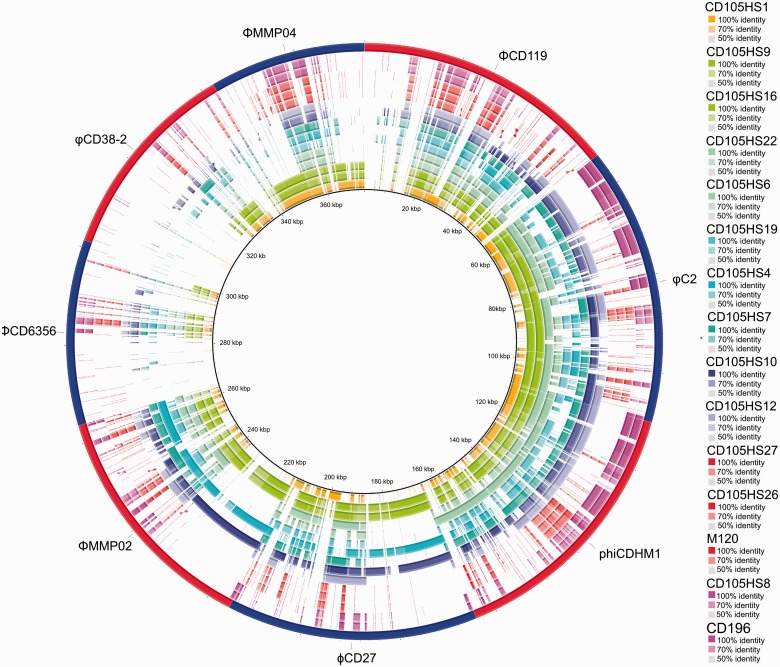
Prophage sequence present across environmental isolates. Composite genome comparison figure generated using BRIG from performing a BLASTn analysis. Rings correspond to each draft genome sequence input for the 13 isolates, CD196 and M120 in the same order as figure 1. The reference sequences used is a multi-FASTA containing genome sequence of the published temperate phage genomes which are shown in alternative red and blue segments in the outermost ring. Color of the ring indicates ribotype and intensity the sequence similarities. Of the 15 genomes searched, 14 have similarity across the lengths of the medium-sized myoviruses, ΦCD119, φC2, and phiCDHM1. In contrast, fewer isolates have sequence similar to the long tailed myoviruses, and the small myovirus and least to each siphovirus. Patterns of the similarity illustrate the conservation of specific regions of the phage genomes, for example, there is less conservation in the structural gene region (at the 3′-end of the genome) of ΦCD119 compared with φC2.

The BRIG analysis shows that there are sequences throughout each draft genome that are similar to known *C. difficile* phages, with the exceptions of the two siphoviruses ΦCD6356 and φCD38-2 (fig. 2). It is important to note that the *C. difficile* phages vary according to their genetic relatedness, which is often reflected by their particle morphology, see review (Hargreaves and Clokie 2014). In brief, the myoviruses can be grouped into the medium myoviruses, which are ΦCD119, φC2 and phiCDHM1 with genome sizes approximately 53–56 kb, capsid diameters approximately 50–60 nm and contractile tails of approximately 100–110 nm (Goh, Riley, et al. 2005; Govind et al. 2006; Hargreaves, Kropinski, et al. 2014); the long-tailed myoviruses, ϕCD27 and ΦMMP02, with genomes approximately 50–52 kb, capsid diameters of 60–65 nm and tail lengths of 210–258 nm (Mayer et al. 2008; Meessen-Pinard et al. 2012); and small myoviruses which is represented by ΦMMP04 with a genome of approximately 32 kb, capsid of approximately 58 nm and tail length of approximately 106 nm (Meessen-Pinard et al. 2012). The siphoviruses are distinct from the myoviruses as well as to one another, with ΦCD6356 has a genome size of approximately 38 kb, capsid diameter or approximately 64 nm and tail length of approximately 272 nm (Horgan et al. 2010) and φCD38-2 has a 41 kb genome, with a capsid diameter of approximately 60 nm and tail length of approximately 210 nm (Sekulovic et al. 2011).

Several of the draft genomes contain more than one distinct type of phage sequence. In order to interpret the results, and assign a specific phage carriage to an isolate, it is important to know the patterns of homology between related phage genomes. For example, the medium myoviruses are genetically similar, with the exception of the structural region of ΦCD119. Although the long-tailed myoviruses are less similar across their lysogeny conversion, control and DNA replication modules, and both types share some homology across these regions as well as the attachment and lysis modules. However, the small myovirus is genetically distinct from the other myoviruses, with some similarity in predicted tail genes and lysis genes, but has greater sequence similarity within its DNA replication region to the siphovirus φCD38-2. The two siphoviruses themselves are distinct from one another, only sharing homologous regions within the lysis and DNA replication regions. The relative higher frequency of these conserved sequences can be observed in the genome comparisons.

Sequences related to the medium-sized myoviruses were the most commonly detected across isolates, with large regions similar to φC2 present in ten of the draft genomes. An example of clear cocarriage of prophage sequences is in the isolates CD105HS10 and CD105HS4 as each has similar sequences to the long-tailed myoviruses ϕCD27 and ΦMMP02, as well as the medium myoviruses ΦCD119, φC2 and phiCDHM1. Sequence similar to ΦMMP04 was detected in CD105HS1, which is the same ribotype as CD630, (R012), but does not carry this prophage. The isolates CD105HS9 and CD105HS16 (R220) have sequences with similarities to all the three myovirus types. In contrast, no isolate appears to contain prophages similar to either of the siphoviruses.

The approach described above relies on available phage sequences, and in order to identify novel prophages, each draft genome was queried using the prophage prediction software PHAST (Zhou et al. 2011). The results of the PHAST analysis confirm the presence of intact as well as fragmented prophages related to the known *C. difficile* phages. The predicted prophage regions not only occur at locations throughout the assembled draft genomes but also frequently are split in contigs, which have been arranged at the ends of the draft genomes; highlighting the difficulty in assembling these regions (data not shown).

In addition to the *C. difficile* phages described above, the prophage in R027 strains CD196 and R20291 is also carried by CD105HS8 (R027) (supplementary fig. S7, Supplementary Material online), and is most similar to that of R20291 (Stabler et al. 2009). Also from the PHAST results, the Tn*6164* of M120 (Corver et al. 2012) was identified in CD105HS27 and CD105HS26 due to the fact that the transposon also contains prophage-like genes in addition to putative antibiotic resistance and DNA replication genes.

**Fig. 3.—:**
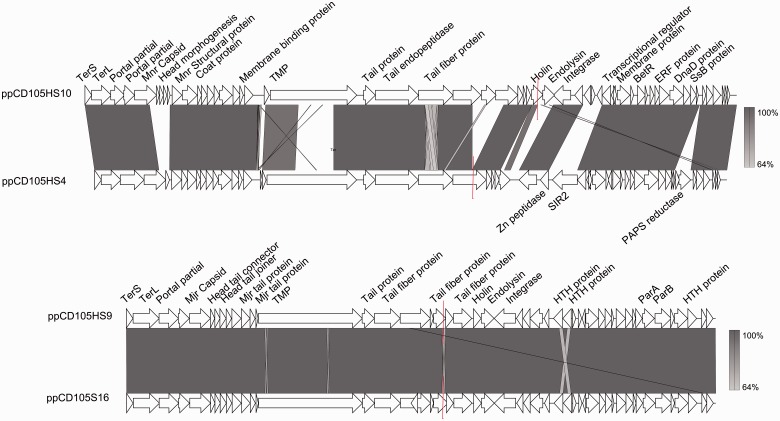
Two novel prophage-like regions containing siphovirus-like CDSs. Linear genome maps of the predicted prophage regions. Top: CD105H9S10 (ppCD105HS10) and CD105HS4 (ppCD105HS4), below: CD105HS16 (ppCD105HS16) and CD105HS9 (ppCD105HS9). Isolates CD105HS10 and CD105HS4 are different ribotypes, R005 and R014, respectively, but CD105HS16 and CD105HS are both R010. The prophage regions have CDSs encoding phage proteins involved in all stages of temperate phage infecting, and are arranged in a similar architecture to the other *C. difficile* phages with terminase, structural, lysis and attachment, lysogeny control, and DNA replication. Color shading indicates sequence similarity through BLASTn, and red lines indicate a break in the region by contig gap.

The analysis also revealed a prophage region containing siphovirus-like genes shared between CD105HS16 and CD105HS9 (both R220), and a second novel region in CD105HS4 and CD105HS10, R014 and R005, respectively (fig. 3). These two novel regions are not present in the other draft genomes, CD196 or M120 (supplementary fig. S8, Supplementary Material online). Each type has recognizable head packaging and morphogenesis genes, structural genes, lysis and attachment, lysogeny control and DNA replication, and metabolism genes (supplementary tables S3 and S5, Supplementary Material online). Phylogenetic analysis showed the predicted endolysin genes each grouped the endolysin genes of the two *C. difficile* siphoviruses and ΦCD119 (supplementary fig. S9, Supplementary Material online).

These two novel prophages are not present in the reference *C. difficile* strains, but similar sequences are present in other *C. difficile* whole-genome shotgun assemblies as revealed by BLAST searches against the NCBI databases. Sequences similar to ppCD105HS16 and ppCD105HS9 are present in the United States and Australian human isolates and include atoxigenic and toxigenic strains from non-CDI carriage and CDI co-colonized patients. Sequences related to ppCD105HS4 and ppCD105HS10 are present in toxigenic isolates from acute CDI and non-CDI carriage patients.

### Phage Resistance and Immunity Mechanisms

One way that *C. difficile* could control phage infection, and the transmission of genetic material transfer through the population, is the CRISPR/Cas (Clustered Regularly Interspaced Palindromic Repeats/CRISPR associated proteins) system (Hargreaves, Flores, et al. 2014). The draft genomes were searched for Cas proteins, with all isolates identified as having at least one set of Cas genes, with genes homologous to the Cas3, Cas5, Cas7 and Cas6 genes, and some isolates having an additional set of genes homologous to Cas2, Cas1, Cas4, Cas3, Cas5, DevR, Cas8 and Cas6, and all isolates contained multiple predicted CRISPR arrays as identified using CRISPR finder (Grissa et al. 2007). In addition to CRISPR/Cas systems, bacteria can have multiple and diverse ways to block or resist phage infection (Labrie et al. 2010). *Clostridium difficile* genomes are known to contain many predicted cell surface proteins and enzymes that process these proteins (Sebaihia et al. 2006), one of which has been shown to be controlled in phase variable manner (Reynolds et al. 2011). There is evidence of several potential mechanisms in the environmental draft genomes, for example, in CD105HS9 there is a putative toxin–antitoxin (TA) system, with a gene homologous to PemK (Pfam PF02452, *E* value 1.5e-32) and an adjacent predicted antitoxin (Agarwal et al. 2010). There is also a predicted abortive infection gene associated with a prophage element, containing an Abi_2 protein domain (Pfam PF07751, *E* value 3.3e-49), several predicted endonucleases, type I and type III restriction enzymes including a homolog of HsdR (Obarska-Kosinska et al. 2008), as well as a predicted 5-methycytosine restriction enzyme containing a McrBC protein domain (PF10117, *E* value 2.7e-79). The functionality of these systems remains to be determined but their carriage is particularly interesting due to the presence of a C-5 cytosine-specific DNA methylase in several *C. difficile* myovirus genomes (e.g., Hargreaves, Kropinski, et al. 2014).

### Phages Infection of Environmental Isolates

**Fig. 4.—:**
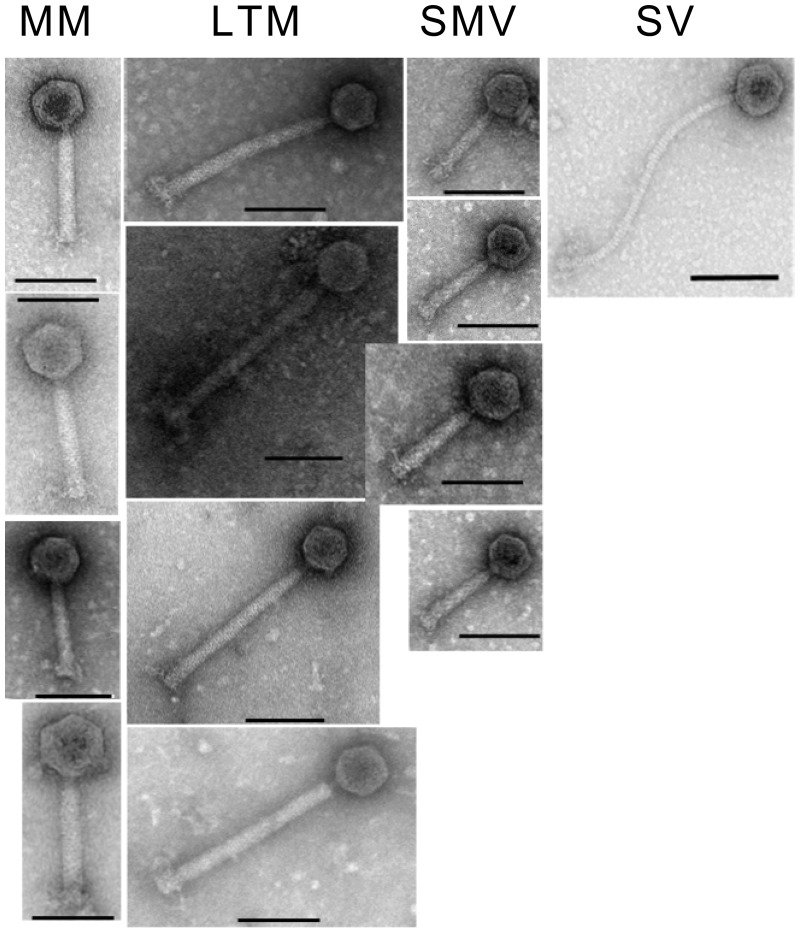
Phage particle morphology. Transmission electron micrographs showing the particle morphologies of the 13 phage; the medium myoviruses (MMs) in descending order; phiCDHM1, phiCDHM3, phiCDHM19 and phiCDHM23, the long tailed myoviruses (LTMS) phiCDHM2, phiCDHM4, phiCDHM5 and phiCDHM6, the small myoviruses (SMV) phiCDHM9, phiCDHM11, phiCDHM13 and phiCDHM14 and siphovirus (SV) phiCDHS1. Scale bar is 100 nm.

To test the susceptibility of these isolates to infection by phages from the same reservoir, phages were isolated from either enriched cultures or induced from these and other environmental strains, as described previously (Hargreaves, Kropinski, et al. 2014). These phages were then tested using spot assays against the isolates sequenced in this study (supplementary table S4, Supplementary Material online). The isolates could all be infected by at least one phage tested, with the exception of both of the R078 isolates. The phages include different types as determined by their particle morphology visualized by TEM (fig. 4). We observed both clear and turbid plaque formation across phages and bacterial isolates. Interestingly, both isolates belonging to R220 showed different phage susceptibility. The induced phage phiCDHM1 from CDH105HS6 was identified in the genome assembly as expected. Our results demonstrate the ability of phages from within this reservoir to infect strains from the same source.

## Discussion

### Whole-Genome Alignment and Sequence Comparison

We applied high-throughput sequencing (HTS) technology to assess the genetic diversity in *C. difficile* strains from an environmental reservoir. HTS has been used to sequence the first *C. difficile* genome (Sebaihia et al. 2006), to determine its epidemiology (Eyre et al. 2012, 2013; He et al. 2013), and to investigate diversity across ribotypes and time (Stabler et al. 2006, 2009, 2012; Cairns et al. 2012). Its genome evolution is marked by the exchange of mobile genetic regions (Sebaihia et al. 2006; Stabler et al. 2009; He et al. 2010, 2013; Monot et al. 2011; Brouwer et al. 2013), and the content of which is thought to reflect its adaptability in response to the different environmental factors such as during growth in the gut system (Sebaihia et al. 2006). Thus the highly variable mobilome highlights one way that this species can persist as an enteric pathogen, as well as facilitate niche adaption (Sebaihia et al. 2006; Stabler et al. 2006). Our analysis is consistent with the view that the genetic flexibility seen within *C. difficile* is driven by such gain or loss of genetic islands [9, 11, 16]. We found that isolates within ribotype groups shared the greatest ANI, and that R078 isolates had the greatest dissimilarity to all other ribotypes. The species is highly diverse with one study finding as little as 19.7% of genes shared by all strains. PCR ribotyping has been found to robustly reflect genomic content across several groups (Kurka et al. 2014). This population of genetically heterogeneous strains may act as a source from which virulent strains emerge as the evolution of virulent lineages appears to have occurred independently, at multiple times (He et al. 2010; Cairns et al. 2012). Typically these evolved lineages are highly clonal, such as R027 (Stabler et al. 2009), but are continuing to evolve in recent time (He et al. 2010; Valiente et al. 2012). The majority of the isolates examined in this study are ribotypes other than those with reference sequences available. The whole-genome alignments in MAUVE before and after reordering contigs to either CD630, or CD196 or M120 resulted in different Local Colinear Blocks (LCB) patterns (data not shown) and highlight the need for more reference strain sequences representing several ribotype groups.

The sequence comparisons performed using BRIG revealed the divergence across the isolates according to the reference genome of CD630. Major regions of absence or dissimilarity occur at sites populated by transposons. This has clear implications for the biology of these strains as it has been established that several conjugative transposons can carry antibiotic resistance genes, for example, *TetM* on Tn*5397* (Mullany et al. 1990), *erm*B on Tn*5398* in CD630 (Mullany et al. 1995), and a chloramphenicol resistance gene on Tn*6103* in CD196 and R20291 (Roberts et al. 2008; Stabler et al. 2009). The exchange of other genetic material can also occur during conjugation events which transfer MGEs, for example, the transfer of the PaLoc was demonstrated alongside the exchange of Tn*5397* between CD630 and an atoxigenic strain CD37 (Brouwer et al. 2013).

Further investigation using ACT with the R078 isolates revealed presence of a putative transposon with genes encoding predicted sporulation proteins and a LexA repressor which contains an HTH_19 (PF12844) at the N terminus and a C terminus Peptidase_S24 (PF00717). LexA is a transcriptional regulator associated with the SOS response that was recently found to have binding sites in the PaLoc and predicted promoters of several sporulation and antibiotic resistance genes (Walter et al. 2014). CD105HS27 has both a chromosomally encoded LexA and the putative LexA on the transposons, which share 23% identity at the aa level so may function differently and impact phenotype. *Clostridium difficile* transposons can excise and transfer between strains (e.g., Brouwer et al. 2011). It remains to be demonstrated whether this transposon can be exchanged but we observed its presence in two publically available sequences of isolates belonging to R126. This ribotype group is highly related to R078; only five core genes were identified which were unique to R078 and not present in R126 isolates (Kurka et al. 2014). In the R078 isolates in our analysis, four of the five CDSs were detected using BLASTn. The presence of this transposon-like element in isolates across both groups suggests that there may be ongoing horizontal gene transfer between these lineages.

Toxin gene content can vary between strains with different content of the PaLoc and binary toxin genes (Rupnik et al. 2009). The PaLoc has a history of multiple acquisition events, exchange, and loss (Dingle et al. 2014). Previously, the toxin gene content of these isolates had been determined using PCR and, where known, those results were mostly confirmed by the BRIG analysis in this study. The BRIG figure highlights the presence of *cdtA* and *cdtB* pseudogenes in several isolates.

Other variable phenotypic trait linked to pathogenicity includes motility, with flagella biosynthesis genes involved in colonization, adherence, and colonization (Tasteyre et al. 2001; Dingle et al. 2011; Baban et al. 2013), in addition to impact upon toxin production (Aubry et al. 2012) and genome-wide gene regulation (Barketi-Klai et al. 2014). Variation has been identified between strains in individual gene sequences, such as within *fliC* (Tasteyre et al. 2000), or entire regional polymorphism, for example, in the F1 and F2 regions of R027 isolates and the deletion of F3 in the nonmotile M120 (Stabler et al. 2009). This region is not present in M120 and is also absent in the draft genomes of the two environmental isolates belonging to R078. We found previously that although one of these isolates was nonmotile and correlates to the genetic information from the draft genome analysis, another, CD105HS26, was motile and the genetic basis of this remains unclear (Hargreaves et al. 2013).

Also highly variable is the content of cell wall proteins (Cwp), which are present in several divergent locus in strain (Biazzo et al. 2013). In particular, the *slpA* which encodes the S-layer protein has a variable region that is highly heterogeneous throughout the species (Fagan and Fairweather 2014). This gene is located on the chromosome adjacent to several homologous genes encoding Cwp proteins in the SlpA locus. The cell surface layer is attributed to bacterial growth and protection against environmental conditions, and the additional Cwp proteins have been linked to functions that maintain the surface layer, cell wall structure, and cell adherence (Reynolds et al. 2011; Fagan and Fairweather 2014). In the SlpA locus, the majority of divergence is present within this gene, but there was an absence of the predicted calcium-binding gene CD_27970 in CD630.

One system that has been shown to be involved in the regulation of virulence and colonization determinants is the *C. difficile* Agr system. Strains can carry different *agr* loci, *agr1-3*, with all strains examined having *agr1*, and *agr3* associated with MGEs as is present near transpose genes and in a temperate phage genome (Stabler et al. 2009; Hargreaves, Kropinski, et al. 2014). An R027 mutant in *agrA* (a transcriptional regulator) of *agr2* was found to decrease its pathogenicity in mice (Martin et al. 2013). Phylogenetic analysis of the *agrB* genes in the environmental strains showed all the three loci present in the population, with *agr3* present in the three R078 genomes and the natural lysogen of phiCDHM1. Whether the different *agr* loci have different functionality and how the third type could impact upon the physiology of R078 remain to be established.

### Identification of Prophage Elements in the Environmental Isolates

The presence of multiple and related prophages in single *C*.* difficile* genomes makes their analysis challenging. We applied two approaches to determine prophage carriage within the draft genomes. The first was to use known phage genomes as references to search the draft genomes through BLASTn. Similarly, earlier studies have used hybridization of DNA probes to identify related phage sequences across *C. difficile* isolates (Goh, Riley, et al. 2005; Fortier and Moineau 2007; Meessen-Pinard et al. 2012). Prophage content has also been estimated by amplification of key phage genes by PCR, such as the holin and capsid genes as markers for prophage carriage (Nale et al. 2012; Shan et al. 2012; Hargreaves et al. 2013). These approaches rely on having an already known phage sequence or sequences to use as references. Although results from such analyses will give information relating to the specific reference phage or phage gene used, it is likely to give only a limited view and underestimate prophage infection due to false negatives. The use of whole-genome comparisons using BLASTn is a bioinformatics approach analogous to DNA-based hybridization which provides resolution at the sequence level. Using BRIG, the prophage content without relying on either the correct assembly or marker genes. There are sequences related to at least one of the known phages in most of the isolates examined, and in many cases to more than one phage type. The predominance of sequences related to the medium-sized myoviruses was evident, which is similar to the finding that 84% of isolates examined containing φC2-like sequence (Goh et al. 2007). As the BRIG analysis also reveals the level of sequence similarity, with the knowledge of phage module or gene locations, it is possible to observe the most conserved and more divergent regions across prevalent phage genomes. We suggest that the use of BRIG is particularly useful when hunting for known phage sequences in draft genome assemblies.

This approach, however, may mask multiple occurrences of related types of prophage as well as identify types different to the reference genomes used. Novel prophage discovery has been performed by TEM following inducing cultures (Fortier and Moineau 2007; Goh et al. 2007; Nale et al. 2012; Hargreaves et al. 2013). This method identifies prophages that are mobilizable and provides information on their morphology, but also is likely to underestimate their prevalence and does not provide genetic information. To complement the BRIG analysis, in this study the prophage prediction tool PHAST was also used to determine prophage content. This approach can confirm the cocarriage of related prophages and miss-assembly of prophage regions from their predicted gene content, and an alternative would also be to assess the relative coverage of these regions in the draft assemblies. This approach relies more heavily on the correct genome assembly and requires manual validation of predictions, but its application led to the identification of the two novel prophage regions for *C. difficile.* These contained several siphovirus-like genes. The phylogenetic analysis of their endolysin genes supports their assignation to this family. Whether these prophages are able to exchange through the population is unknown but in the previous study, siphovirus-like particles were observed from two of the isolates’ culture lysates using TEM (Hargreaves et al. 2013). This approach also provides a means of discovering new genes that have the potential to be developed for the treatment of *C. difficile*, such as the purified endolysin of ϕCD27 which has been investigated as novel therapeutic (Mayer et al. 2008).

As all but one isolate in this study were previously investigated for prophage release and *C. difficile* phage marker genes [7], we compared these results with the BRIG and PHAST predictions generated in this study. There were clear correlations, but also cases where the results did not agree fully, and we suggest that a combination of approaches is most useful while working toward understanding prophage diversity within this species. For example, siphovirus-like particles were observed in culture lysates from CD105HS4 and CD105HS16, which carry the novel prophage types. However, in contrast, particles were not observed for CD105HS9 or CD105HS10, which we know from our bioinformatic data, also contain these novel prophages [7]. This confirms the previously acknowledged limitations of using TEM to examine for presence of phages and suggests that there may be several instances where there may be cryptic prophage elements in strains may be common but only observable from sequence data.

When considering phage use as a potential alternative therapy against *C. difficile*, it is important to realize the potential inhibition of phage infection through superinfection with related phages (Govind et al. 2011). The distribution of prophage types in this data set may explain some of the observations from bacterial host range data using multiple phage panels. The phage φCD38-2 has been reported to have a wide host range and was least frequently detected in this data set, contrasting with the other phages that have typically exhibited a narrow spectrum of infectivity, and a larger proportion of the strains harboring medium myovirus-like sequences was found (Mayer et al. 2008; Horgan et al. 2010; Sekulovic et al. 2011, 2014). We tested the phage susceptibility to a panel of 13 phages, representing four main types according to particle morphology and genome size. These include medium myoviruses, long tailed myoviruses, small myoviruses, and a siphovirus. All isolates could be infected by at least one phage, with the exception of the R078 isolates. There was no clear correlation between prophage carriage type and infected phage morphology.

Our results show that a highly lysogenic population of *C. difficile* is present, with types of recognizable phages and novel types carried by different isolates. Several *C. difficile* phages have been found to both modulate bacterial toxin production (Goh, Chang, et al. 2005; Meader et al. 2010; Sekulovic et al. 2011) and carry genes that could alter virulence (Hargreaves, Kropinski, et al. 2014). The phage φC2 has been demonstrated to transfer genetic material through transduction (Goh et al. 2013), it seems likely that, if active in this reservoir, the mixed prophage content within population could have a significant impact on the *C. difficile* biology. Multiple resistance mechanisms exist which that could control phage infection within the species, and remain to be experimentally determined.

To conclude, whole-genome sequencing of 13 *C. difficile* environmental isolates provides important genetic level insights in the biology of strains in this reservoir. This study has used different bioinformatic approaches to assess strain diversity at a whole-genome view. The degree of variance between strains is particularly characterized within regions associated with mobile elements, and we have identified multiple carriage both of known *C. difficile* temperate phages and novel prophage types. We know that several of *C. difficile* prophages are able to excise, and several strains carry prophages closely related to φC2 which has been experimentally shown to transfer genetic material between *C. difficile* strains. These findings support the hypothesis that environmental reservoirs of *C. difficile* contain strains that are highly related to clinical settings, and demonstrate that they have a genetically diverse associated mobilome. Whether ongoing evolution is occurring within these populations or prior to their entry has not been established and is the subject of future research in our laboratory.

## Supplementary Material

Supplementary tables S1–S6 and figures S1–S9 are available at *Genome Biology and Evolution* online (http://www.gbe.oxfordjournals.org/).

Supplementary Data
