# Supplementary material for: As Clear as Mud? Determining the Diversity and Prevalence of Prophages in the Draft Genomes of Estuarine Isolates of Clostridium difficile
Source: Genome Biol Evol. 2015 May 27;7(7):1842–55. doi: 10.1093/gbe/evv094 (PMC4524475; doi:10.1093/gbe/evv094)
Supplement: Supplementary Data [file supp_7_7_1842__index.html]

As clear as mud? Determining the diversity and prevalence of prophages in the draft genomes of estuarine isolates of Clostridium difficile. — As Clear as Mud? Determining the Diversity and Prevalence of Prophages in the Draft Genomes of Estuarine Isolates of Clostridium difficile — Supplementary Data 

# As Clear as Mud? Determining the Diversity and Prevalence of Prophages in the Draft Genomes of Estuarine Isolates of *Clostridium difficile*

## Supplementary Data

files

- Supplementary Data - docx file
- Supplementary Data - docx file
