# Supplementary material for: As Clear as Mud? Determining the Diversity and Prevalence of Prophages in the Draft Genomes of Estuarine Isolates of Clostridium difficile
Source: Genome Biol Evol. 2015 May 27;7(7):1842–55. doi: 10.1093/gbe/evv094 (PMC4524475; doi:10.1093/gbe/evv094)
Supplement: Supplementary Data [file supp_evv094_New_Microsoft_Office_Word_Document.docx]

**Figure S1. Genome blastn comparisons of R078 isolates.**

Composite genome comparison figure generated using BRIG from performing a blastn analysis for each of the R078 isolate as reference with; left M120 as reference with on the inner most ring CD105HS27 (red) and outermost ring CD105HS26 (red), middle CD105HS27 as reference with innermost ring M120 (black) and outermost ring CD105HS26 (red) and right CD105HS26 as reference with M120 (black) and CD106HS27 (red). Region of major divergence is a novel transposon-like element.

**Figure S2. Transposon-like element in environmental R078 isolates.**

Map of transposon-like element in R078 isolates CD105HS27 (middle) and CD105HS26 (top), with blastn analysis and insert region in M120 (bottom). Black arrows indicate conserved CDSs and white arrows the CDSs present in the transposon-like element. The element is flanked by a predicted integrase and site specific recombinase (labelled). The transposon-like element is entire in CD105HS27 but fragments in CD105HS26, with a partial region assembled to its corresponding location in CD105HS27, and additional sequence has assembled at the end of the draft genome as can be seen in Figure S1.

**Figure S3. PaLoc and CDT gene sequences in environmental *C. difficile* whole draft genomes.**

Composite genome comparison figure generated using BRIG from performing a blastn analysis for each of the 13 isolates, strains CD196 and M120 to reference genes of the PaLoc, *tcdA*, *tcdE*, *tcdB*, *tcdR* and *tcdC*, the transcriptional regulatory *cdtR* from CD630 and binary toxin encoding genes *cdtA* and *cdtB* from CD196. On rings from the inside to the outside tracks are; CD105HS1 (R012), CD105HS9 (R010), CD105HS16 (R010), CD105HS22 (R220), CD105HS6 (R220), CD105HS19 (R031), CD105HS4 (R014), CD105HS7 (R002), CD105HS10 (R005), CD105HS12 (R001), CD105HS27 (R078), CD105HS26 (R078), M120 (R078), CD105HS8 (R027), CD196 (R027) and gene features of interest from CD630 annotation which are labelled. The colour correlates to ribotype group, e.g. R078 are red and R027 are magenta and intensity to sequence similarity %. Gapped regions indicate the absence or low similarity between the genomes and genes. It is evident that there are genes absent or present as well as pseudogene copies within the draft genomes with three atoxigenic strains identified, two are R010 and one R031. 10 of the isolates do not have complete copies of the binary toxin genes and the three atoxigenic isolates also do not have *cdtR*.

**Figure S4. Blastn comparison of Flagella regions of environmental isolates.**

Composite genome comparison figure generated using BRIG from performing a blastn analysis for each of the 13 isolates, strains CD196 and M120 to genes involved in flagella biosynthesis from reference CD630. On rings from the inside to the outside tracks are; CD105HS1 (R012), CD105HS9 (R010), CD105HS16 (R010), CD105HS22 (R220), CD105HS6 (R220), CD105HS19 (R031), CD105HS4 (R014), CD105HS7 (R002), CD105HS10 (R005), CD105HS12 (R001), CD105HS27 (R078), CD105HS26 (R078), M120 (R078), CD105HS8 (R027), CD196 (R027), genes of the region with gene names labelled and outermost ring arcs indicate the F1, F2 and F3 regions. The ring colour correlates to ribotype group. The F1 region is the most conserved across all draft genomes, in contrast to the F2 region which is only present in the isolate belonging to R012. The F3 region appears to be missing in all three R078 isolates; CD105HS27 and CD106HS27 and M120.

**Figure S5. Blastn comparison of SlpA locus of environmental isolates.**

Composite genome comparison figure generated using BRIG from performing a blastn analysis for each of the 13 isolates, strains CD196 and M120 to genes in the SlpA locus includes *slpA* and several cell surface proteins such as *cwp84*. On rings from the inside to the outside tracks are; CD105HS1 (R012), CD105HS9 (R010), CD105HS16 (R010), CD105HS22 (R220), CD105HS6 (R220), CD105HS19 (R031), CD105HS4 (R014), CD105HS7 (R002), CD105HS10 (R005), CD105HS12 (R001), CD105HS27 (R078), CD105HS26 (R078), M120 (R078), CD105HS8 (R027), CD196 (R027), genes of the region with gene names labelled. The ring colour correlates to ribotype group. There is considerable relative variation in the *slpA* across the isolates compared to the CD630 sequence, and in some upstream genes. It also appears that the gene encoding the predicted calcium binding adhesion protein is missing in the three R078 isolates; CD105HS27 and CD106HS27 and M120.

**Figure S6. Phylogenetic analysis of *agrB* indicating *agr* loci presence across environmental *C. difficile* isolates.**

Maximum likelihood analysis was performed on the aligned amino acid sequences identified in the draft genomes in MAUVE. The clusters reflect the *agr* loci types present in the species, with all 16 taxa having a copy of *agrB* from *agr1*, seven taxa also have a copy of *agrB* from *agr2* and four taxa have a copy of *agrB* from *agr3* locus including the three R078 isolates and an isolate of R220 which is a lysogen containing phiCDHM1*.* Branches of the tree are highlighted by colour to indicate the R078 (red) and R027 (magenta) groups which cluster with strong bootstrap support.

**Figure S7. Blastn comparison of prophage of R027 isolates.**

The prophage region in CD105HS8 (on middle line) is most similar to that in R20291 (on top line) compared to that in CD196 (on bottom line) due to the short divergent region in the DNA replication and metabolism region. The prophage regions have been re-orientated to begin with the TerS gene and analysed using blastn in EasyFig v2.1

**Figure S8. Prophages ppCD105HS10 and ppCD105HS16 sequence distributed across environmental isolates.**

Composite genome comparison figure generated using BRIG from performing a blastn analysis for each of the 13 isolates, strains CD196 and M120, to the prophages ppCD106HS10 and ppCD106HS16. On rings from the inside to the outside tracks are; CD105HS1 (R012), CD105HS9 (R010), CD105HS16 (R010), CD105HS22 (R220), CD105HS6 (R220), CD105HS19 (R031), CD105HS4 (R014), CD105HS7 (R002), CD105HS10 (R005), CD105HS12 (R001), CD105HS27 (R078), CD105HS26 (R078), M120 (R078), CD105HS8 (R027), CD196 (R027). The ring colour correlates to ribotype group.

**Figure S9. Phylogeny of *C. difficile* phage and novel prophage endolysins**

Maximum likelihood analysis was performed on the aligned amino acid sequences of the predicted endolysin genes from the published *C. difficile* phages, and the novel prophage regions identified in this study in order to determine their relatedness. The two genes from the related prophages in CD105HS10 and CD105HS4 cluster to the two *C. difficile* siphoviruses with strong bootstrap support, however the genes from the other two prophages ppCD105HS9 and ppCD105HS16 cluster to that of the medium myovirus ΦCD119, despite being highly dissimilar across the rest of the genome (data not shown). The aa sequence of the endolysin from the *Clostridium* siphovirus phiSM101 was used as an outgroup to build the tree.
