# Supplementary material for: As Clear as Mud? Determining the Diversity and Prevalence of Prophages in the Draft Genomes of Estuarine Isolates of Clostridium difficile
Source: Genome Biol Evol. 2015 May 27;7(7):1842–55. doi: 10.1093/gbe/evv094 (PMC4524475; doi:10.1093/gbe/evv094)
Supplement: Supplementary Data [file supp_evv094_Supplementary_Tables.docx]

Table S1. Average nucleotide identity between *C. difficile* genome assemblies.

|  | CD105HS1 | CD105HS4 | CD105HS6 | CD105HS7 | CD105HS8 | CD105HS9 | CD105HS10 | CD105HS12 | CD105HS16 | CD105HS19 | CD105HS22 | CD105HS26 | CD105HS27 | CD630 | CD196 | M120 |
| --- | --- | --- | --- | --- | --- | --- | --- | --- | --- | --- | --- | --- | --- | --- | --- | --- |
| CD105HS1 | 100.0 | 99.4 | 99.4 | 99.3 | 99.0 | 99.4 | 99.3 | 99.4 | 99.4 | 99.4 | 99.4 | 96.5 | 96.4 | 99.8 | 99.0 | 96.7 |
| CD105HS4 |  | 100.0 | 99.8 | 99.4 | 99.1 | 99.4 | 99.4 | 99.5 | 99.4 | 99.5 | 99.8 | 96.6 | 96.4 | 99.5 | 99.1 | 96.8 |
| CD105HS6 |  |  | 100.0 | 99.4 | 99.1 | 99.5 | 99.4 | 99.5 | 99.5 | 99.5 | 99.9 | 96.6 | 96.5 | 99.5 | 99.1 | 96.8 |
| CD105HS7 |  |  |  | 100.0 | 99.0 | 99.4 | 99.4 | 99.4 | 99.4 | 99.4 | 99.4 | 96.5 | 96.4 | 99.4 | 99.0 | 96.7 |
| CD105HS8 |  |  |  |  | 100.0 | 99.1 | 99.0 | 99.0 | 99.1 | 99.1 | 99.1 | 96.6 | 96.4 | 99.9 | 99.1 | 96.7 |
| CD105HS9 |  |  |  |  |  | 100.0 | 99.4 | 99.5 | 99.9 | 99.5 | 99.5 | 96.6 | 96.4 | 99.5 | 99.0 | 96.7 |
| CD105HS10 |  |  |  |  |  |  | 100.0 | 99.5 | 99.4 | 99.4 | 99.4 | 96.5 | 96.4 | 99.4 | 99.0 | 96.7 |
| CD105HS12 |  |  |  |  |  |  |  | 100.0 | 99.5 | 99.5 | 99.5 | 96.6 | 96.4 | 99.5 | 99.1 | 96.7 |
| CD105HS16 |  |  |  |  |  |  |  |  | 100.0 | 99.5 | 99.5 | 96.6 | 96.4 | 99.4 | 99.1 | 96.7 |
| CD105HS19 |  |  |  |  |  |  |  |  |  | 100.0 | 99.5 | 96.6 | 96.4 | 99.1 | 99.5 | 96.7 |
| CD105HS22 |  |  |  |  |  |  |  |  |  |  | 100.0 | 96.6 | 96.4 | 99.5 | 99.1 | 96.7 |
| CD105HS26 |  |  |  |  |  |  |  |  |  |  |  | 100.0 | 99.3 | 96.5 | 96.6 | 99.6 |
| CD105HS27 |  |  |  |  |  |  |  |  |  |  |  |  | 100.0 | 96.4 | 96.4 | 99.5 |
| CD630 |  |  |  |  |  |  |  |  |  |  |  |  |  | 99.9 | 99.1 | 96.7 |
| CD196 |  |  |  |  |  |  |  |  |  |  |  |  |  |  | 99.9 | 96.8 |
| M120 |  |  |  |  |  |  |  |  |  |  |  |  |  |  |  | 99.8 |

Table S2. Predicted CDS features of the transposon-like element in CD105HS27.

| Aa size | Strand orientation | | Gene name | Product and evidence from Pfam and TIG | | | | | |  | | |  |
| --- | --- | --- | --- | --- | --- | --- | --- | --- | --- | --- | --- | --- | --- |
| 262 | + | soj_2 | | sporulation initiation inhibitor | | | | | |  | | |  |
| 310 | + |  | | stage 0 sporulation protein DNA-binding protein | | | | | | | | |  |
| 104 | + |  | | hypothetical protein | | |  | | |  | | |  |
| 133 | + |  | | hypothetical protein | | |  | | |  | | |  |
| 133 | + | ssb_2 | | single-strand DNA-binding protein | | | | | | | | |  |
| 131 | + |  | | hypothetical protein | | |  | | |  | | |  |
| 308 | + |  | | replication initiation protein | | | | | |  | | |  |
| 271 | + |  | | replication initiation protein | | | | | |  | | |  |
| 266 | + | dnaC_1 | | DNA replication protein DnaC | | | | | |  | | |  |
| 164 | + |  | | conjugative transposon protein | | | | | |  | | |  |
| 642 | + |  | | conjugative transfer protein | | | | | |  | | |  |
| 272 | + |  | | membrane protein | | |  | | |  | | |  |
| 818 | + |  | | Hydrolase |  | |  | | |  | | |  |
| 927 | + |  | | cell wall hydrolase | | |  | | |  | | |  |
| 107 | + |  | | hypothetical protein | | |  | | |  | | |  |
| 460 | + |  | | cell surface protein | | |  | | |  | | |  |
| 187 | + |  | | transcriptional activator, adenine-specific DNA methyltransferase | | | Cdd | | | COG4725 | | |  |
| 691 | + |  | | DNA topoisomerase III | | | | | |  | | |  |
| 1400 | + |  | | cell surface protein | | |  | | |  | | |  |
| 733 | + |  | | cell surface protein | | |  | | |  | | |  |
| 362 | + |  | | hypothetical protein | | |  | | |  | | |  |
| 104 | + |  | | hypothetical protein | | |  | | |  | | |  |
| 214 | + | radC_2 | | DNA repair protein RadC | | | | | |  | | |  |
| 164 | + |  | | DNA/RNA helicase | | |  | | |  | | |  |
| 2404 | + |  | | DNA/RNA helicase | | |  | | |  | | |  |
| 487 | + |  | | ABC transporter ATP-binding protein | | | | | | | | |  |
| 255 | + |  | | peptidase C60B | | |  | | |  | | |  |
| 1034 | + |  | | anti-restriction protein | | | | | |  | | |  |
| 276 | + |  | | hypothetical protein | | |  | | |  | | |  |
| 121 | + |  | | hypothetical protein | | |  | | |  | | |  |
| 307 | + |  | | conjugative transposon protein | | | | | |  | | |  |
| 75 | + |  | | conjugative transposon protein | | | | | |  | | |  |
| 289 | - | aadK_1 | | aminoglycoside 6-adenylyltransferase | | | | | | | | |  |
| 210 | - |  | | acetoin dehydrogenase E2 subunit dihydrolipoyllysine-residue acetyltransferase | | | CLUSTERS | | | PRK14875 | | |  |
| 267 | - |  | | HTH-type transcriptional regulator | | | | | | | | |  |
| 138 | + |  | | conjugative transposon protein | | | | | |  | | |  |
| 315 | + |  | | Plasmid recombination enzyme | | | Pfam | | PF01076.13 | | | |  |
| 541 | + | tndX_1 | | recombinase site-specific resolvase family protein | | | | | | | | |  |
| 532 | + | mobA_2 | | DNA strand transferase | | | | | |  | | |  |
| 188 | + | dnaG_1 | | DNA primase | | |  | | |  | | |  |
| 442 | + |  | | putative P-loop ATPase | | | Cdd | | | COG5545 | | |  |
| 239 | + |  | | conjugative transfer protein | | | | | |  | | |  |
| 627 | + |  | | reverse transcriptase/maturase/endonuclease | | | | | | | | |  |
| 407 | - |  | | putative transporter | | | CLUSTERS | | | PRK10054 | | |  |
| 542 | + |  | | endonuclease relaxase | | | | | |  | | |  |
| 140 | + |  | | phage replisome organizer, putative, N-terminal region | |  | | TIGRFAM | | | TIGR01714 | | |
| 463 | + | tndX_2 | | recombinase site-specific resolvase family protein | | | | | | | | |  |
| 278 | + | rlmA | | 23S rRNA (guanine(745)-N(1))-methyltransferase | | | | | | | | |  |
| 182 | - |  | | hypothetical protein | | |  | | |  | | |  |
| 465 | + |  | | endonuclease relaxase | | | | | |  | | |  |
| 640 | + |  | | DNA repair protein RadA | | | CLUSTERS | | | PRK11823 | | |  |
| 89 | + |  | | conjugative transposon protein | | | | | |  | | |  |
| 250 | + |  | | cell surface protein | | |  | | |  | | |  |
| 679 | + | topB_2 | | DNA topoisomerase III | | | | | |  | | |  |
| 145 | + |  | | conjugative transposon protein | | | | | |  | | |  |
| 107 | + |  | | hypothetical protein | | |  | | |  | | |  |
| 1003 | + |  | | anti-restriction protein | | | | | |  | | |  |
| 70 | + |  | | conjugative transposon protein | | | | | |  | | |  |
| 445 | - |  | | endonuclease relaxase | | | | | |  | | |  |
| 110 | - |  | | mobilization protein | | |  | | |  | | |  |
| 119 | - |  | | HTH-type transcriptional regulator | | | | | | | | |  |
| 328 | + |  | | radical SAM-family protein | | | | | |  | | |  |
| 304 | + | tndX_3 | | recombinase site-specific resolvase family protein | | | | | | | | |  |
| 456 | - |  | | endonuclease relaxase | | | | | |  | | |  |
| 108 | - |  | | mobilization protein | | |  | | |  | | |  |
| 120 | + |  | | hypothetical protein | | |  | | |  | | |  |
| 157 | + |  | | hypothetical protein | | |  | | |  | | |  |
| 270 | + |  | | hypothetical protein | | |  | | |  | | |  |
| 405 | + |  | | Transposase | | Cdd | | | | COG3328 | | |  |
| 247 | - |  | | LexA repressor | | CLUSTERS | | | | PRK00215 | | |  |
| 416 | + | dinB_1 | | DNA polymerase IV | |  | | | |  | | |  |
| 80 | + |  | | hypothetical protein | |  | | | |  | | |  |
| 191 | + |  | | phage transcriptional regulator, ArpU family | | TIGRFAM | | | | TIGR01637 | | |  |
| 122 | + |  | | hypothetical protein | | |  | | |  | | |  |
| 105 | + |  | | HTH-type transcriptional regulator | | | | | | | | |  |
| 73 | + |  | | Sporulation initiation factor Spo0A C terminal | | | | | | | |  |  |
| 140 | + |  | | hypothetical protein | | |  | | |  | | |  |
| 158 | + |  | | sigma factor | | |  | | |  | | |  |
| 128 | + | EndoA_1 | | endoribonuclease toxin | | | | | |  | | |  |
| 83 | + |  | | hypothetical protein | | |  | | |  | | |  |
| 564 | + | tndX_4 | | Recombinase site-specific resolvase family protein | | | | | | | | |  |
| 564 | + | tndX_5 | | Recombinase site-specific resolvase family protein | | | | | | | | |  |
| 526 | + | tndX_6 | | Recombinase site-specific resolvase family protein | | | | | | | | |  |

Table S3. Antibiotic resistance of isolates against erythromycin and tetracycline.

| Isolate | Ery | Tet |  | ARDB prediction | | | |
| --- | --- | --- | --- | --- | --- | --- | --- |
| CD105HS9 | + | - | ant6ia | baca | ermb |  |  |
| CD105HS16 | + | - | ant6ia | baca | ermb |  |  |
| CD105HS22 | + | - |  | baca |  |  |  |
| CD105HS6 | - | - |  | baca |  |  |  |
| CD105HS27 | + | + | ant6ia | baca | ermb | tet32 | tetm |
| CD105HS26 | - | + | ant6ia | baca |  | tet40 | tetm |
| CD105HS8 | + | - |  | baca |  |  |  |
| CD105HS1 | + | - | aac6ie | baca |  |  |  |
| CD105HS19 | - | - |  | baca |  |  |  |
| CD105HS4 | - | - |  | baca |  |  |  |
| CD105HS7 | - | - |  | baca |  |  |  |
| CD105HS10 | - | - |  | baca |  |  |  |
| CD105HS12 | - | - |  | baca |  |  |  |

Ery = Erythormycin 20 µgml^-1^ ,Tet = tetracylcine 10 µgml^-1^, + = growth, - = no growth, ant6ia and aac6ie = aminoglycoside resistance, baca = bacitracin resistance, ermb = erythromycin resistance, tet32, tet 40 and tetM =

Table S5. Annotation table for ppCD105HS10 from blastp against NCBI nt/nr virus (taxid ID 10239) database.

| Aa length | Strand | Product | | E value | % identity | Highest scoring blastp result | morphology |
| --- | --- | --- | --- | --- | --- | --- | --- |
| 146 | + | Terminase SSU | 5.00E-39 | | 51% | *Thermus* phage phiOH2 | Siphovirus |
| 421 | + | Terminase LSU | 0 | | 98% | *Paenibacillus* phage Davies, Jimmer1, Jimmer2 and Abouo | Myovirus |
| 295 | + | portal protein (partial) | 3.00E-18 | | 76% | *Clostridium* phage phiCTP1 | Siphovirus |
| 230 | + | portal protein (partial) | 3.00E-16 | | 80% | *Clostridium* phage phiCTP1 | Siphovirus |
| 472 | + | minor head protein | 3.00E-41 | | 40% | *Streptococcus* phage Dp-1 | Siphovirus |
| 58 | + | hypothetical protein |  | |  |  |  |
| 79 | + | minor head protein | 1.00E-07 | | 41% | *Lactobacillus* phage phi jlb1 | Myovirus |
| 71 | + | hypothetical protein |  | |  |  |  |
| 58 | + | hypothetical protein |  | |  |  |  |
| 211 | + | scaffold structural protein | 3.00E-13 | | 28 | *Lactococcus* phage BM13 | Siphovirus |
| 322 | + | phage coat protein | 3.00E-106 | | 52 | *Enterococcus* phage phiFL3A, *Enterococcus* phage phiFL3B | Siphovirus |
| 84 | + | hypothetical protein |  | |  |  |  |
| 122 | + | hypothetical protein |  | |  |  |  |
| 144 | + | hypothetical protein | 1.00E-23 | | 43 | *Clostridium* phage phiCD27 | Myovirus |
| 114 | + | hypothetical protein |  | |  |  |  |
| 268 | + | hypothetical protein | 5.00E-33 | | 36 | *Clostridium* phage phiCTP1 | Siphovirus |
| 158 | + | hypothetical protein | 3.00E-14 | | 30 | *Clostridium* phage phiCTP1 | Siphovirus |
| 77 | + | hypothetical protein | 6.00E-08 | | 40 | *Clostridium* phage phiCTP1 | Siphovirus |
| 180 | + | hypothetical protein | 7.00E-07 | | 70 | *Enterococcus* phage phiEf11 | Siphovirus |
| 109 | + | hypothetical protein |  | |  |  |  |
| 1856 | + | TMP | 1.00E-56 | | 54 | *Clostridium* phage phiMMP02 | Myovirus |
| 251 | + | tail component | 5.00E-47 | | 39 | *Clostridium* phage phiCD38-2 | Siphovirus |
| 922 | + | tail/endopeptidase | 4.00E-158 | | 58 | *Clostridium* phage phiCD38-2 | Siphovirus |
| 740 | + | tail fiber | 3.00E-32 | | 29 | *Clostridium* phage phiCD38-2 | Siphovirus |
| 302 | - | hypothetical protein |  | |  |  |  |
| 596 | + | tail fiber | 1.00E-176 | | 49 | *Clostridium* phage phiCD38-2 | Siphovirus |
| 315 | + | tail fiber | 1.00E-26 | | 40 | *Clostridium* phiCD27 | Myovirus |
| 477 | + | tail fiber | 7.00E-03 | | 33 | *Lactobacillus* phage Lb338-1 | Myovirus |
| 105 | + | hypothetical protein |  | |  |  |  |
| 56 | + | hypothetical protein |  | |  |  |  |
| 88 | + | Holing | 6.00E-50 | | 93 | *Clostridium* phage phiCD3802 | Siphovirus |
| 376 | - | Integrase | 1.00E-163 | | 64 | *Clostridium* phage phiC2 | Myovirus |
| 168 | - | hypothetical protein | 3.00E-08 | | 34 | *Listeria* phage LP-030-2 | Siphovirus |
| 140 | - | hypothetical protein |  | |  |  |  |
| 73 | + | hypothetical protein | 2.00E-19 | | 63 | *Staphylococcus* phage X2 and 187 | Siphovirus |
| 108 | - | hypothetical protein | 2.00E-09 | | 37 | *Staphylococcus* phage X2 | Siphovirus |
| 181 | - | Repressor | 2.00E-28 | | 79 | *Clostridium* phage C2 | Myovirus |
| 86 | - | hypothetical protein |  | |  |  |  |
| 71 | + | cro-like repressor | 1.00E-19 | | 75 | *Clostridium* phiC2 | Myovirus |
| 271 | + | anti-repressor | 6.00E-70 | | 45 | *Listeria* phage B054 | Siphovirus |
| 60 | + | hypothetical protein |  | |  |  |  |
| 165 | + | hypothetical protein | 3.00E-24 | | 39 | *Clostridium* phage phiC2 | Myovirus |
| 49 | + | hypothetical protein |  | |  |  |  |
| 80 | + | hypothetical protein | 7.00E-05 | | 48 | *Clostridium* phage phiC2 | Myovirus |
| 93 | + | hypothetical protein |  | |  |  |  |
| 213 | + | essential recombination protein | 1.00E-96 | | 69 | *Clostridium* phage CDHM1 | Myovirus |
| 287 | + | phage replication protein | 3.00E-16 | | 31 | *Clostridium* phage phiCD27 | Myovirus |
| 139 | + | sDNA binding protein | 5.00E-49 | | 58 | *Clostridium* phiCD6356 | Siphovirus |
| 120 | + | hypothetical protein | 1.00E-45 | | 66 | *Clostridium* phage phiCD27 | Myovirus |
| 44 | + | hypothetical protein | 4.00E-22 | | 95 | *Clostridium* phage phiC2 | Myovirus |
| 66 | + | hypothetical protein |  | |  |  |  |
| 138 | + | hypothetical protein | 2.00E-29 | | 46 | *Staphylococcus* phage 69 | Siphovirus |
| 166 | + | sigma factor | 2.00E-65 | | 63 | *Clostridium* phiMMP02 | Myovirus |
| 39 | + | hypothetical protein |  | |  |  |  |
| 43 | + | hypothetical protein |  | |  |  |  |
| 58 | + | hypothetical protein | 8.00E-09 | | 47 | *Clostridium* phiCD38-2 | Siphovirus |

Table S4. Annotation table for ppCD105HS16 from blastp against NCBI nt/nr virus (taxid ID 10239) database

| Aa length | Strand | Product | | E value | % identity | Highest scoring blastp result | morphology |
| --- | --- | --- | --- | --- | --- | --- | --- |
| 141 | + | TerS | 3-e10 | | 30 | *Streptococcus* phage phi-m46.1 and phi-SsUD.1 | NA |
| 559 | + | TerL | 0 | |  | *Clostridium* phage phiS63 | Siphovirus |
| 417 | + | portal | 7.00E-170 | |  | *Geobacillus* virus E2 | Siphovirus |
| 235 | + | portal | 7.00E-170 | | 33 | *Geobacillus* virus E2 | Siphovirus |
| 448 | + | Mjr Capsid | 1.00E-120 | | 51 | *Clostridium* phage phiSM101 |  |
| 91 | + | DNA packaging protein | 1.00E-16 | | 55 | *Bacillus* phage phiS3501 | Siphovirus |
| 112 | + | head-tail joining protein | 1.00E-11 | | 43 | *Paenibacillus* phage phiBB_PI23 | Siphovirus |
| 131 | + | hypothetical protein |  | |  |  |  |
| 113 | + | hypothetical protein |  | |  |  |  |
| 196 | + | Mjr tail protein | 4.00E-07 | |  | *Clostridium* phage phiSM101 |  |
| 184 | + | hypothetical protein |  | | 81 |  |  |
| 109 | + | hypothetical protein |  | | 33 |  |  |
| 49 | + | hypothetical protein |  | |  |  |  |
| 2227 | + | TMP | 2.00E-111 | |  | *Clostridium* phage phiCD6356 | Siphovirus |
| 281 | - | hypothetical protein |  | |  |  |  |
| 260 | + | hypothetical protein | 4.00E-11 | | 97 | *Bacillus* phage SPBc2 | Siphovirus |
| 554 | + | tail protein | 5.00E-25 | |  | *Clostridium* phage c-st | Myovirus |
| 648 | + | tail fiber | 1.00E-05 | | 49 | *Bacillus* phage vB BceM-Bc431v3 | Myovirus |
| 142 | - | hypothetical protein |  | |  |  |  |
| 66 | + | hypothetical protein |  | | 31 |  |  |
| 302 | + | hypothetical protein | 9.00E-09 | | 88 | *Clostridium* phage phiCP39-O | Siphovirus |
| 149 | + | hypothetical protein |  | |  |  |  |
| 389 | + | hypothetical protein |  | |  |  |  |
| 165 | + | Holing | 3.00E-06 | | 34 | *Streptococcus* phi-SsUD.1 |  |
| 274 | + | Endolysin | 9.00E-65 | | 29 | *Clostridium* phage phiMMP02 | Myovirus |
| 457 | - | Integrase | 8.00E-154 | |  | *Clostridium* phage phiCD27 | Myovirus |
| 134 | - | hypothetical protein | 2.00E+31 | |  | *Clostridium* phage phiCD119 | Myovirus |
| 143 | - | hypothetical protein |  | | 37 |  |  |
| 276 | - | hypothetical protein |  | | 37 |  |  |
| 103 | - | hypothetical protein |  | | 37 |  |  |
| 191 | - | HTH protein | 1.00E-13 | | 30 | *Clostridium* phage phiCD6356 | Siphovirus |
| 179 | - | HTH protein | 4.00E-08 | |  | *Clostridium* phage phiCD6356 | Siphovirus |
| 72 | + | hypothetical protein |  | |  |  |  |
| 71 | + | hypothetical protein |  | | 33 |  |  |
| 62 | - | hypothetical protein |  | | 51 |  |  |
| 260 | + | hypothetical protein | 8.00E-62 | | 55 | *Clostridium* phage phiS63 | Siphovirus |
| 137 | + | hypothetical protein |  | | 43 |  |  |
| 151 | + | hypothetical protein | 2.00E-06 | |  | *Clostridium* phage CDMH1 | Myovirus |
| 43 | + | hypothetical protein |  | |  |  |  |
| 200 | + | recombination protein | 2.00E-05 | |  | *Bacillus* phage BceA1, *Bacillus* phage Waukesha92, *Staphylococcus* phage SpaA1 | Siphovirus, NA, Siphovirus |
| 150 | + | hypothetical protein | 4.00E-04 | | 81 | *Staphylococcus* phage StB12 | Siphovirus |
| 62 | + | hypothetical protein |  | | 33 |  |  |
| 141 | + | hypothetical protein |  | |  |  |  |
| 251 | + | ParA | 4.00E-45 | |  | unidentified phage | NA |
| 346 | + | ParB | 6.00E-25 | |  | unidentified phage | NA |
| 76 | + | hypothetical protein |  | | 97 |  |  |
| 280 | + | hypothetical protein |  | |  |  |  |
| 190 | + | hypothetical protein | 7.00E-27 | | 49 | *Paenibacillus* phage Emery | Myovirus |
| 108 | + | hypothetical protein | 5.00E-07 | |  | *Clostridium* phage phiCD6356 | Siphovirus |
| 143 | + | HNH endonuclease | 2.00E-21 | | 31 | *Clostridium* phage phiSM101 |  |

Table S7. Phage susceptibility of environmental isolates.

| Ribotype | Isolate | phiCDHM1 | phiCDHM2 | phiCDHM3 | phiCDHM4 | phiCDHM5 | phiCDHM6 | phiCDHM9 | phiCDHM11 | phiCDHM13 | phiCDHM14 | phiCDHM19 | phiCDHM23 | phiCDHS1 |
| --- | --- | --- | --- | --- | --- | --- | --- | --- | --- | --- | --- | --- | --- | --- |
| R010 | CD105S16 |  |  |  |  |  |  |  |  |  |  |  |  | ○ |
|  | CD105HS9 |  |  |  |  |  |  |  |  |  |  |  |  | ○ |
| R002 | CD105HS7 | ◙ |  |  |  |  |  |  |  |  |  |  |  |  |
| R031  R012 | CD105HS19 |  |  | ○ |  |  |  |  | ◙ | ○ | ○ |  |  |  |
|  | CD105HS1 |  | ○ |  | ○ |  |  |  | ○ | ○ | ○ |  |  |  |
| R005 | CD105HS10 |  |  |  |  |  |  |  |  |  |  |  |  | ○ |
| R220 | CD105HS22 |  |  |  |  | ◙ | ◙ |  |  |  |  |  |  |  |
|  | CD105HS6 |  |  |  |  | ○ | ○ | ○ | ○ |  |  |  |  |  |
| R014 | CD105HS4 |  |  |  |  | ○ | ○ |  |  |  |  |  |  |  |
| R001  R027 | CD105HS12 |  |  |  |  |  |  |  |  |  |  |  |  | ○ |
|  | CD105HS8 |  |  |  |  |  | ○ |  |  |  |  | ○ | ○ |  |
| R078 | CD105HS26 |  |  |  |  |  |  |  |  |  |  |  |  |  |
|  | CD105HS27 |  |  |  |  |  |  |  |  |  |  |  |  |  |

Symbol ○ indicates lytic infection and ◙ indicates turbid infection.
